# *Mycobacterium avium* ssp. *paratuberculosis* in the Food Supply: A Public Health Issue

**DOI:** 10.3389/fpubh.2021.647448

**Published:** 2021-07-15

**Authors:** Lauren Kuenstner, John Todd Kuenstner

**Affiliations:** ^1^Department of Health and Human Services, Center for Medicare and Medicaid Innovation, Windsor Mill, MD, United States; ^2^Department of Medicine, Temple University Lewis Katz School of Medicine, Philadelphia, PA, United States

**Keywords:** *Mycobacterium avium* ssp *paratuberculosis*, milk pasteurization, zoonosis, Crohn's disease, food safety regulations, FDA food safety modernization act, pasteurized milk ordinance, dairy products

## Abstract

This article examines the policy implications of *Mycobacterium avium* subspecies *paratuberculosis* (MAP) as a zoonotic pathogen and the public health risks posed by the presence of MAP in food, particularly milk products. Viable MAP has been cultured from commercially pasteurized milk in the US. Dairy pasteurization standards and regulations are examined in light of this finding. On the basis of the precautionary principle, the authors suggest options to reduce exposure to MAP, including (1) increased federal authority to regulate pasteurization of all dairy products, (2) modification of pasteurization standards in order to more effectively kill MAP, (3) removal of the Pasteurized Milk Ordinance (PMO) provision that allows states to override federal policy in intrastate dairy sales, and (4) creation of a mandatory Johne's Disease Control Program. These measures would reduce human exposure to MAP and may reduce the risk of diseases associated with MAP.

## Introduction: *Mycobacterium avium* Subspecies *Paratuberculosis* and Johne's Disease

*Mycobacterium avium* subspecies *paratuberculosis* (MAP) is a bacterium that causes Johne's disease (JD) in ruminants including cattle, sheep, goats, deer, bison, llamas, and elk (1). JD primarily infects the intestine and results in a gradually progressive disease, including poor digestion, prolonged diarrhea, and excessive weight loss (2, 3). In advanced stages of disease, animals may become excessively weak and die (3, 4). Cows infected with MAP excrete the organism in their feces and, to a lesser extent, in milk. The bacterium is excreted into the milk within the udder, but MAP may also enter raw milk through fecal contamination during milking (3, 5).

Although MAP has historically been considered purely an animal pathogen, a growing body of evidence suggests that MAP is a zoonotic pathogen, raising challenges for policymakers and public health officials to ensure the safety of the food supply. This article discusses the role of MAP as a zoonotic pathogen, the presence of the organism in the food supply, particularly in milk, and state and federal regulations relating to dairy products and pasteurization. Options to reduce the level of MAP in milk products are suggested. Although MAP has also been cultured from beef and some sources of municipal water, the focus of this article is the presence of MAP in milk and ways to reduce this presence.

### MAP: A Zoonotic Pathogen

MAP was first reported in the US in 1908 (4) and has since been considered solely an animal pathogen. Thomas Kennedy Dalziel first speculated in 1913 that Crohn's disease (CD), a human disease mirroring JD, is caused by MAP (6). While a wide range of etiologies have been proposed for CD (7, 8), a growing body of evidence implicates MAP as a zoonotic pathogen. However, because this body of evidence is still developing, insufficient attention has been paid to the presence and containment of MAP in dairy and beef herds in the US outside of the agricultural and veterinary communities.

Research beginning in the 1980s suggests that MAP may be a zoonotic pathogen. Chiodini first reported culturing MAP from the intestinal tissues of CD patients in 1984 (9). In 2004, Naser et al. cultured MAP from the blood of 50% of patients with CD (10). Meta-analyses by Feller (11) and Abubakar (12) concluded that a majority of the studies on the association of MAP with CD demonstrate that most patients with CD are infected with MAP.

In 2017, an international group of clinicians and researchers convened to discuss the science of MAP and human disease. A majority of the attendees concluded that the body of evidence strongly supports the theory that MAP is a zoonotic bacterium causing CD and a public health threat (13).

Subsequently, a study of CD patients and non-CD control subjects was conducted and demonstrated the presence of viable MAP in the blood of CD patients with a significantly increased odds ratio, but also demonstrated the viable MAP organism in some of the non-CD controls (14). The study employed four different MAP culture methods and four MAP antibody tests, included 201 subjects (61 CD patients and 140 non-CD controls) and was conducted in five laboratories (14).

DY Graham has reported good supportive evidence that MAP causes human disease in his recent abstract describing the results of the RedHill BioPharma, FDA phase 3, controlled clinical trial of combination anti-MAP antibiotics for CD (15).

In a case series report, Kuenstner et al. documented several patients diagnosed with autoimmune diseases, and CD, who were infected with MAP (16). Two of these patients were treated with anti-MAP therapy and subsequently were shown to be free of MAP infection and disease (16). This case series report supports the pathogenicity of MAP in humans (16). Additionally, a case series report by Agrawal et al. showing profound remission in 10 CD patients treated with anti-MAP therapy provides further support to the theory that MAP causes human disease (17).

### Viable MAP in the Milk Supply

A growing body of evidence now exists that suggests that MAP is a zoonotic pathogen, and therefore, the presence of viable MAP in commercially available pasteurized milk in the US is a serious public health issue (18–21).

There are a number of methods available to reduce the incidence of MAP in dairy products designed for human consumption. One commonly held view is that pasteurization is an effective way to destroy MAP. Pasteurization can be achieved through a number of different methods and has been employed as a public health measure for over 100 years in the US. These include Higher-Heat Shorter-Time (HHST), ultra-pasteurization (UP), and ultrahigh temperature (UHT). The most commonly used form of pasteurization in the US is the High-Temperature Short-Time method (HTST pasteurization) (18).

While HTST is widely accepted as an effective method to destroy pathogens present in raw milk (19), a recent study has demonstrated HTST's ineffectiveness at inactivating all MAP naturally present in milk. Grant et al. cultured viable MAP from 6.9% of HTST-pasteurized milk samples in Ireland (20). On the basis of their findings, Grant et al. concluded that MAP is capable of surviving commercial HTST pasteurization if it is present in raw milk in sufficient numbers (20). While this study examined samples from the UK, the method of pasteurization utilized was the same as the US standard, implying that commercially pasteurized dairy products in the US may also contain viable MAP despite pasteurization.

Retail pasteurized milk samples from Wisconsin, Minnesota, and California were found to contain viable MAP (21). Ellingson et al. collected 702 pints of commercially pasteurized whole milk from three of the top five milk-producing states. Viable MAP was detected in 2.8% of the samples (21). The presence of viable MAP was not limited to a particular retail brand: over half of the brands (12 of 22, or 55%) of retail pasteurized whole milk contained at least one sample positive for viable MAP (21). Additionally, the National Advisory Committee on Microbiological Criteria for Foods, affiliated with the USDA Food Safety and Inspection Service (FSIS), acknowledged that milk, particularly raw milk, is a likely source of MAP exposure (22).

### U.S. Dairy Pasteurization Standards

U.S. dairy pasteurization standards are administered primarily via the Pasteurized Milk Ordinance (PMO), FDA, and states. The PMO is widely accepted as the national standard for milk sanitation in the US (23, 24). In 1987, the FDA mandated “the pasteurization of all milk and milk products in final package form for direct human consumption for all milk shipped in interstate commerce” (25). This mandate also applies to imported milk and milk products. The FDA prohibits the importation of unpasteurized milk and milk products (26). As a result of these regulations, raw milk may only be distributed across state lines if it is en route to plants to be pasteurized or used to make aged cheese before being sold to customers (26, 27). Some hard cheeses are exempt from pasteurization (19). Cheese makers have contended that aging cheese for 60 days or longer kills pathogens; this claim is being reevaluated by the FDA (27).

### Limits of the Current Regulatory Framework

Because the FDA/USPHS is only vested with jurisdiction over interstate commerce under the Public Health Service Act (28) and the Federal Food, Drug and Cosmetic Act (29), it cannot regulate intrastate commerce (24). The USPHS/FDA can only enforce milk sanitation standards on interstate carriers and milk products shipped in interstate commerce (23); therefore, the FDA has limited power to address public health issues such as MAP in the milk supply. Accordingly, the FDA may only recommend that states, counties, and municipalities legally adopt the PMO to encourage higher standards of milk sanitation practices and greater public health law uniformity (23). The FDA recommends that all of the PMO be adopted and that “no changes be made to the Ordinance when adopted by a State or Local community, unless changes are necessary to avoid conflict with State law” (23). Additionally, the FDA warns that modifications to the Ordinance should be considered with extreme caution in order to preserve the enforceability of the law. Once the Ordinance has been adopted locally, its enforcement becomes the responsibility of Local and/or State authorities.

The PMO discourages the use of state public health laws to create unnecessary trade barriers between states and/or municipalities. All 50 states, the District of Columbia, and U.S. Trust Territories participate in the voluntary Cooperative State-USPHS/FDA Program for the Certification of Interstate Milk Shippers, which relies on the PMO (23).

Forty-six states have adopted all, or many, of the provisions of the Ordinance (26). California, Maryland, New York, and Pennsylvania have not adopted the PMO and have enacted similar milk safety laws instead (26). While the PMO appears to foster nationally uniform pasteurization laws, key components weaken its enforceability, encouraging fragmentation of the national dairy safety system.

A fundamental flaw in the PMO is the allowance for state statutes, administrative codes, or policies to override federal regulation in the event of conflict between state and federal law. Thus, states can legalize raw milk sales or distribution through three channels:

1. Statute: any state statute conflicting with section 9 of the PMO overrides the Ordinance,
2. Administrative regulation or code: any state regulation conflicting with section 9 of the PMO overrides it, and
3. Policy: State policy can override state statutes and administrative rules in the event of conflict (26).

Even in a state that prohibits the sale of raw milk, state regulatory agencies may decide not to shut down cow-share programs if they comply with state guidelines, permitting the sale of raw milk (26). Consequently, despite the adoption of the PMO by 46 states, it remains legal for an individual to access raw milk for human consumption in 39 states (26) (Table 1). Of the states that permit raw milk for human consumption, many prohibit sales of most or all raw milk products, including butter and cheese (26). The remaining 11 states prohibit raw milk sales (26).

**Table 1:** Raw milk access across the United States.

**State**	**Raw milk in retail stores**	**Raw milk at farmer's markets**	**Raw milk on farm**	**Raw milk *via* cowshare program**	**Raw milk on farm (goat only)**	**Prohibit all raw milk**
Alabama						X
Alaska				X		
Arizona	X					
Arkansas			X			
California	X					
Colorado				X		
Connecticut	X					
Delaware						X
District of Columbia						X
Florida						X
Georgia						X
Hawaii						X
Idaho	X					
Illinois			X			
Indiana				X		
Iowa						X
Kansas			X			
Kentucky					X	
Louisiana						X
Maine	X					
Maryland						X
Massachusetts			X			
Michigan				X		
Minnesota			X			
Mississippi					X	
Missouri		X				
Montana						X
Nebraska			X			
Nevada	X					
New Hampshire	X					
New Jersey						X
New Mexico	X					
New York			X			
North Carolina						X
North Dakota				X		
Ohio				X		
Oklahoma			X			
Oregon			X			
Pennsylvania	X					
Rhode Island					X	
South Carolina	X					
South Dakota			X			
Tennessee				X		
Texas			X			
Utah	X					
Vermont			X			
Virginia				X		
Washington	X					
West Virginia				X		
Wisconsin			X			
Wyoming		X				
Total (including D.C.)	12	2	13	9	3	12

*National conference of state legislatures, state milk laws*.

The enactment and implementation of the FDA Food Safety Modernization Act (FSMA) in 2011 did not address this public health issue. While the FSMA authorizes unprecedented expansions to the FDA's authority, it does not incorporate policies recognizing MAP as a zoonotic pathogen and maintains the current HTST standard (30). The first controlled clinical trial of anti-MAP therapy for CD is supportive of the theory that MAP causes human disease and with this information, the FDA should act to prevent foodborne illness according to its mandate (31).

Current laws regulating the pasteurization of milk should be reevaluated.

### Estimates of Milk-Borne Illness in the US

In light of the studies implicating MAP as a cause of CD, access to raw milk in 39 of 50 states is concerning. CD is debilitating, costly, chronic, and potentially fatal. CD affects ~780,000 people in the US (32), representing an estimated economic burden of up to $11–28 billion a year in direct costs (in conjunction with ulcerative colitis) (32). Earlier estimates excluding ulcerative colitis found $10.9–15.5 billion in US annual direct costs attributed to CD (33).

Under the current regulatory framework, the public is exposed to MAP in both unpasteurized and pasteurized milk and dairy products. While the CDC acknowledges that occasional pasteurized dairy product disease outbreaks do occur, the CDC contends that these illnesses are generally mild compared to more severe illnesses acquired through raw dairy products, rendering pasteurized dairy products mostly safe (34). The FDA estimates that milk and fluid milk products account for <1% of all food and waterborne illnesses (23).

Based on the increasing evidence that MAP is a zoonotic pathogen transmitted through the food supply, the CDC, USPHS, and FDA underestimate the frequency and severity of foodborne illness associated with pasteurized dairy products. This renders the US food safety regulatory system insufficient in preventing infectious disease. Even if the entire country adopted the current PMO in full, with no modifications to the law, severe disease outbreaks associated with consumption of pasteurized dairy products would still occur. The PMO recommends HTST pasteurization, which is ineffective in killing all MAP present in raw milk (20).

### Ways to Reduce the Prevalence of MAP in Milk Products

The two primary methods to decrease the presence of MAP in the food supply are (1) upstream—preventing MAP from entering the milk supply through programs to prevent and control MAP infection in dairy herds, and (2) downstream—more effective pasteurization.

Herd prevalence of JD has been estimated at 91.1% nationally (35). The Voluntary Bovine Johne's Disease Control Program (VBJDCP) is the official control program in the US. Almost every state has some form of JD control program. While the USDA Animal and Plant Health Inspection Service recommends uniform standards, the voluntary program has been insufficient in reducing MAP prevalence in dairy cattle due to the low producer awareness of MAP, the program, and the perceived lack of benefit to herd owners. Despite financial incentives offered by the USDA to producers and veterinarians to participate, farmer knowledge of MAP and program participation are still well-below 100% nationally in dairy and beef operations (36, 37).

The absence of a federal JD control mandate within the dairy industry weakens producer participation, endangering herd and financial security, and public health. It is likely that only a mandatory, uniform, and federally administered JD control program will result in 100% farmer participation. Permitting the states to develop and implement their own standards will undoubtedly result in a fragmented JD control system, as evidenced by fragmented milk safety laws permitted by a weak PMO. Under the current regulatory framework, consumers living in a state with weak JD control laws face increased risk of MAP exposure; a federal JD control mandate would lessen that risk.

In conjunction with prevention programs to reduce the prevalence of MAP in cattle, adequate pasteurization processes and regulations may decrease the presence of MAP in the food supply. The scientific community needs to determine adequate pasteurization standards to eliminate viable MAP from retail pasteurized milk and milk products. The public health system could adopt appropriate pasteurization processes once the scientific community has determined the best pasteurization processes for killing MAP.

## Recommendations

The medical literature on human disease and MAP continues to grow but food safety regulations and standards have remained unchanged. If the MAP theory is validated in clinical trials, and scientists agree that one of the modes of transmission is via pasteurized milk products, then as many as 780,000 Americans are living with preventable foodborne illnesses. The cost of MAP in combination with other conditions may be up to $11–28 billion annually (32). In a cost-conscious healthcare and fiscal climate, lawmakers should be receptive to policy measures intended to prevent costly and devastating diseases. The federal government has an incentive to avoid spending on treatment for preventable illnesses. However, it is unclear how much spending that would be avoided by preventing MAP-caused conditions would be offset by increases in federal spending to create a modernized public health infrastructure to minimize MAP exposure.

The following recommendations, if incorporated into federal and state policies, would reduce the risk from MAP:

1. Revise the PMO to federally mandate adequate pasteurization standards (those that decrease or ideally eliminate viable MAP) for all interstate milk and milk products intended for final human consumption.
2. Remove section 9 of the PMO, preventing state/local policy from overriding federal policy in intrastate dairy sales.
3. Expand FDA/USPHS authority to enforce the updated PMO, even in intrastate milk sales.
4. Create laws prohibiting the milking of cattle with demonstrable MAP bacteria in their milk and the sale of artisanal cheese made from raw milk.
5. Establish and fund a mandatory, uniform, USDA-administered JD Control Program.

If the FDA is not vested with the necessary authority to regulate intrastate milk sales, then the U.S. Congress may be able to pass legislation authorizing the FDA to enforce pasteurization of milk and milk products in intrastate commerce. Additionally, if the USDA is not authorized to enforce a mandatory JD Control Program under current federal statutes, then Congress may be able to pass legislation authorizing the USDA to create and enforce this program. If, however, the USDA is authorized to create such a program, then it should consult with various state programs and industry stakeholders to determine best practices for JD prevention and control. These best practices could then be incorporated into the federal program. A mandatory federal JD Control Program will require increased veterinary training in JD, increased farmer education, and increased funding to educate both veterinarians and producers regarding MAP.

The FDA would not be subject to increased authority to mandate recalls, because it is already authorized to do so under the FSMA. The FSMA authorizes the FDA to order mandatory recalls of all FDA-regulated foods other than infant formula suspected of adulteration or misbranding (38). If the FDA suspects adulteration or misbranding and the manufacturer refuses to cease distribution or recall that product, the Secretary of Health and Human Services can order a mandatory recall (38). Consequently, if the FDA were to suspect that retail milk and milk products had been contaminated with MAP, they could require sellers to remove it from shelves and require manufacturers to cease distributing the product and impose fees on violating parties.

Given the number of changes that would be made to the public health infrastructure to reduce MAP exposure to the consumer, consolidation of these recommendations into a single legislative instrument, similar to the FSMA of 2011, could be considered. The intent behind the FSMA and the proposed bill is the same: to reduce the risk of foodborne illness.

Because such action is likely to take a number of years, more immediate action to minimize public exposure to MAP could be taken in the meantime. Realistic softer policies could include, for example,

1. Encouraging state agricultural departments to persuade as many dairy owners to participate in the Voluntary Bovine Johne's Disease Control Program as possible, and offering additional financial incentives to participate, if possible.
2. Encouraging milk processors to voluntarily raise the minimum temperature threshold for pasteurization until the scientific community develops revised standards.

Recently, more food producers have begun to adopt voluntary JD control practices, indicating that softer MAP control policies may provide a viable interim solution.

## Discussion

The presence of MAP in the food supply and its association with CD presents a challenge for policymakers and public health officials to invoke the precautionary principle in the interest of public health. The precautionary principle states that “complete evidence of a potential risk is not required before action is taken to mitigate the effects of the potential risk” (39). Weir et al. offer a framework to guide policymakers and public health officials in the application of the precautionary principle (39). Weir et al. believe that application of the precautionary principle is appropriate when: “(1) the exposure or harm is widespread, (2) the incidence of the harm (i.e., the observed health effect) is increasing and is otherwise unexplained, (3) the suspected harm associated with the exposure is serious, and (4) the suspected harm associated with the exposure is not easily treatable or reversible,” among other criteria (39). By applying the precautionary principle, the public health risk of MAP would be appropriately reduced by implementing the public health interventions discussed in this article.

## Author Contributions

All authors listed have made a substantial, direct and intellectual contribution to the work, and approved it for publication.

## Conflict of Interest

JK has proprietary interests in MAP therapies or diagnostic tests. The remaining author declares that the research was conducted in the absence of any commercial or financial relationships that could be construed as a potential conflict of interest.
